# Athletics

**Published:** 1860-07

**Authors:** 


// HALL'S JOURNAL OF HEALTH.

Our Legitimate Scope is almost boundless: for whatever begets pleasurable and harmless feelings, promotes Health; and whatever induces disagreeable sensations, engenders Disease.

WE AIM TO SHOW HOW DISEASE MAY BE AVOIDED, AND THAT IT IS BEST, WHEN SICKNESS COMES, TO TAKE NO MEDICINE WITHOUT CONSULTING A PHYSICIAN.

Vol. VII.] JULY, 1860. [No. 7.

### ATHLETICS.

NEW-YORK is destined to be one of the grandest cities in the civilized world; because it has within it the elements of mechanical and commercial greatness possessed by none other on the globe. The only obstacles at present seen, which can blast these anticipations, are the want of physical and moral health. The latter may, with great confidence, be committed to an educated, active and devoted clergy, and a consistent, liberal and zealous church membership: as to the former, there are signs of promise; thanks to the intelligent and persevering efforts of Drs. Griscom, Sayers, E. Y. Robbins and a few others, public attention is strongly turning towards securing clean streets, clean houses, clean cellars, clean yards and well-ventilated buildings for the accommodation of the poor. In due time, cheap bathing-houses for the masses; elevated, large, light and airy work-rooms for those who live by sewing, knitting and embroideries, the use of which may be obtained for a few cents a day, thus giving them warmth, pure air, quiet and cleanliness, enabling them to work in comfort, cheerfulness, and health. These things will follow in due time, with others of a like humanizing and benevolent character.

We look with great delight in another direction for facilities to promote the health of the children, the boys and the girls, the young men and maidens of our growing city; that is, to the CENTRAL PARK, one of the very largest for purely public purposes in the world.

Fresh and full of sunshine are the memories of what we have seen on the Paseos of Havanna and Matanzas on the sea, in the garden of the Tuileries, the splendid Champs Elysees and the Parks of London, of thousands of well-dressed people on horse, on foot, in carriage, bent on some gladsome enjoyments, some to see, others to be seen; and all brimful of animation, activity, and life; these daily cavalcades of wealth and fashion and youth and beauty, will of themselves add more to the desirableness of the Metropolis as a residence, than any one other thing connected with it; they will promote health, banish ennui, increase enjoyment, and scatter money, transferring it from the coffers of the liberal rich, to the pockets of the industrious poor and the enterprising, thus increasing the pleasure and happiness of all.

The Central Park will be a magnificent gymnasium, where opportunities will be afforded for every variety of exhilarating exercise. There will be no vain beatings of the air, no frog-puffing, no pendulations of doleful dumb-bells, no vulgar fisticuffs, no objectless arm-swingings; but there will be the drive, the saddle, the promenade, the row, the skate, the cricket and the base-ball. There will be a five-mile foot-path for the lounger, the lover and the contemplative; there will be a nine-mile carriage-drive over an easy graded and beautifully smooth turnpike and a separate bridle-road of twenty miles, now up-hill, now down, straight a bit, then winding around some projecting rock or miniature mountain, through shaded dell and by secluded cavern, over hills and through tunnels, thus giving to our sons and daughters invaluable opportunities for perfecting themselves in that most exhilarating and health-giving of all the graces, riding on horseback.

For this good time coming and soon to be here, we look longingly; for Gotham will then be the Mecca of the sick, the very paradise of doctors, the maelstrom of apothecaries and all druggery! These are not inconsistent ends; they logically concatenate; the ratiocination is perfect, the reasoning is without a flaw. In the first place, the sick will congregate here from all points of the compass for the unequaled (in the whole world) facilities for securing all forms of exercise in the open air, without the disadvantages of dangerous exposures; and as judicious out-door activities, with waking up entertainments, is the million of remedies out of a million and one for the cure of ordinary ailments, the people, through the influence of common-sense and HALL'S JOURNAL OF HEALTH, will at once be aroused to the advantages offered. The sick being once brought here, New-York will be the very elysium of the educated physician, because he will have nothing to do but sit in his office during certain specified hours daily, give advice and take the fees, which last will be liberal, as travelers always have money; and travel has the effect of liberalizing expenditures; for any reader of reflection must know that he does not think one tenth part as much of a dollar abroad as he does at home.

